# Revolutionizing Bladder Health: Artificial-Intelligence-Powered Automatic Measurement of Bladder Volume Using Two-Dimensional Ultrasound

**DOI:** 10.3390/diagnostics14161829

**Published:** 2024-08-22

**Authors:** Evan Avraham Alpert, Daniel David Gold, Deganit Kobliner-Friedman, Michael Wagner, Ziv Dadon

**Affiliations:** 1 Department of Emergency Medicine, Eisenberg R&D Authority, Shaare Zedek Medical Center, Jerusalem 9112001, Israel; avraham.alpert@mail.huji.ac.il (E.A.A.);; 2 Faculty of Medicine, Hebrew University of Jerusalem, Jerusalem 9190500, Israel; 3 Division of Hospital Medicine, Department of Medicine, Prisma Health Greenville Memorial Hospital, 701 Grove Rd, Greenville, SC 29605, USA; 4 Jesselson Integrated Heart Center, Eisenberg R&D Authority, Shaare Zedek Medical Center, Jerusalem 9112001, Israel

**Keywords:** ultrasonography, point-of-care ultrasound, emergency departments, artificial intelligence, bladder outlet obstruction, cauda equina syndrome

## Abstract

Introduction: Measuring elevated post-void residual volume is important for diagnosing urinary outflow tract obstruction and cauda equina syndrome. Catheter placement is exact but painful, invasive, and may cause infection, whereas an ultrasound is accurate, painless, and safe. Aim: The purpose of this single-center study is to evaluate the accuracy of a module for artificial-intelligence (AI)-based fully automated bladder volume (BV) prospective measurement using two-dimensional ultrasound images, as compared with manual measurement by expert sonographers. Methods: Pairs of transverse and longitudinal bladder images were obtained from patients evaluated in an urgent care clinic. The scans were prospectively analyzed by the automated module using the prolate ellipsoid method. The same examinations were manually measured by a blinded expert sonographer. The two methods were compared using the Pearson correlation, kappa coefficients, and the Bland–Altman method. Results: A total of 111 pairs of transverse and longitudinal views were included. A very strong correlation was found between the manual BV measurements and the AI-based module with r = 0.97 [95% CI: 0.96–0.98]. The specificity and sensitivity for the diagnosis of an elevated post-void residual volume using a threshold ≥200 mL were 1.00 and 0.82, respectively. An almost-perfect agreement between manual and automated methods was obtained (kappa = 0.85). Perfect reproducibility was found for both inter- and intra-observer agreements. Conclusion: This AI-based module provides an accurate automated measurement of the BV based on ultrasound images. This novel method demonstrates a very strong correlation with the gold standard, making it a potentially valuable decision-support tool for non-experts in acute settings.

## 1. Introduction

In diverse healthcare settings, particularly in the emergency department, point-of-care ultrasounds (POCUS) prove invaluable. It has been incorporated as an integral assessment, diagnostic, and patient-management tool with multiple applications [1]. While the first use of POCUS was for the focused assessment of sonography in trauma (FAST), the field has expanded to include applications for musculoskeletal, testicular, ocular, cardiac, and resuscitative ultrasounds [2]. In addition to diagnostic purposes, ultrasounds are used by clinicians for procedural guidance. This includes central venous line insertion and regional anesthesia [3,4]. Besides physicians, POCUS in the emergency department are being used by physician assistants and nurses [5]. One foundational application is for aiding intravenous line insertion in patients with difficult access [6]. This has helped improve the first-attempt success rate and facilitated faster insertion times [7]. More advanced applications are being implemented by nurses, but these are usually performed by those with specialized certifications. This includes the ability of specialized nurses to evaluate the volume status in outpatients at a heart failure clinic, as well as facilitating liver biopsies by advanced nurse practitioners [8,9]. However, most nurses do not have this level of advanced training.

The role of the emergency department nurse is either clinical, administrative, or in triage. While triaging patients in the emergency department can be a complex process, with multiple modes of assessment taking place simultaneously, a timely fashioned POCUS assessment can be utilized to facilitate decision making and lead the patient along the correct care path [10,11,12].

Bladder volume measurement is a common procedure with clinical and therapeutic implications for patients diagnosed with urinary dysfunction. Bladder volume can be measured either invasively, with a urinary catheter, or by non-invasive means, such as with two-dimensional (2D) ultrasound devices or dedicated bladder scanners. Bladder volume measurement by a urethral catheter is very accurate but is an invasive, painful procedure that may result in a catheter-associated urinary tract infection, a significant healthcare-associated infection, as well as other urological complications [13,14]. Also, as not all medical team members may have the autonomy to order a urethral catheter insertion, an alternate objective quantitative assessment is required [15]. Ultrasound devices, due to their availability, low cost, and noninvasive nature, have been long recognized as an accurate, safe, and painless alternative for bladder evaluation and bladder volume measurement [16,17].

Ultrasound devices play a pivotal role in measuring bladder volume to determine if there is an elevated post-void residual volume suggestive of a potential functional or physical obstruction, and in confirming an indication to catheterize the patient. Urinary retention, often due to benign prostatic hyperplasia in men or pelvic organ prolapse in women, can also result from infections, medications, and, more urgently, cauda equina syndrome [18]. This warrants decompression of the bladder by the insertion of an indwelling urinary catheter. Since catheter placement is invasive and may lead to unfavorable outcomes, it is recommended to reduce unnecessary catheterizations by first measuring the bladder volume with POCUS to confirm the diagnosis [19]. The exact threshold of residual volume indicating the need for catheterization is not agreed upon in the literature, but it is generally accepted that a post-void residual volume of up to 150 mL is normal [20,21]. A recent prospective study tested the diagnostic performance of ultrasound-based bladder volume assessments on 260 patients who presented with symptoms suspicious of cauda equina syndrome, all assessed by board-eligible spinal surgeons, and who had transabdominal ultrasound bladder scans for pre- and post-void residual volume measurements before a lumbosacral MRI [22]. The study demonstrated that a post-void residual volume of ≥200 mL has a sensitivity of 94.1% and a negative predictive value of 98% for the diagnosis of cauda equina syndrome, allowing for risk stratification with 13% of patients initially deemed ‘low-risk’.

Nonetheless, 2D ultrasound bladder volume measurement can be time-consuming and involves border tracings and the use of formulas not readily available on portable devices, resulting in a possible error-prone procedure that is dependent on the user’s experience. Also, some of the less-experienced medical personnel may need to use expensive designated bladder scanners that are less mobile, not readily available in all clinics and medical departments, and provide a volume estimation with no visual reassurance, especially in early bladder scan models. Inaccuracies of bladder scans were previously shown in cases where morbid obesity or abdominal fluid (e.g., ascites, retroperitoneal hemorrhages) were present [23]. Artificial intelligence (AI) and computational technologies are being widely applied in diverse imaging modalities and across multiple disciplines, aiding non-expert operators in decision making and improving diagnostic capabilities. As such, employing an AI-based tool for bladder volume calculation could be a beneficial and a more appealing alternative in the context of emergency care, obviating the necessity for expensive bladder scan utilization or the implementation of a non-trivial formula [24,25]. Despite different commercially available AI-based algorithms, designed to automatically or semi-automatically calculate the volumetry of the urinary bladder, there is a dearth of data validating these tools in real-life settings.

The objective of this study is to evaluate the accuracy of an AI-based module for fully automated pre- and post-void bladder diameter and volume measurements using 2D ultrasound images by comparing its performance to manual measurements taken by expert sonographers.

## 2. Materials and Methods

### 2.1. Study Setting

This is a single-center study of bladder volume measurement that was based on ultrasound images previously acquired from patients assessed in an outpatient urgent care clinic. The study was approved by the Institutional Review Board of the Shaare Zedek Medical Center (IRB; 014017-SZMC) with a waiver of informed consent.

### 2.2. Study Endpoint

The purpose of this study is to evaluate the accuracy of the AI-based fully automated module for bladder volume measurement, as well as the three diameters of the bladder, using two-dimensional ultrasound images compared with the manual measurements taken by experienced sonographers.

### 2.3. Study Protocol

The images were acquired from adult patients aged 18 years or older, as part of routine abdominal ultrasound examinations performed on an elective basis for a myriad of referral indications by qualified sonography technicians using DC-8 (Mindray, Shenzhen, China) and S2000 (Siemens Healthcare, Estates, IL, USA) ultrasound devices. The studies were performed in the supine posture with the head of the bed flat and when possible, with flexed knees. Scans included (at minimum) a liver view, kidney views, and bladder views. Data were collected in the Digital Imaging and Communications in Medicine (DICOM) format. All participants underwent abdominal ultrasound examinations that consisted of bladder volume measurements and a complete set of bladder images (i.e., longitudinal and transverse views of the bladder). For examinations that included two different sets of post-voiding and pre-voiding images, both sets were separately included in the assessment. Tests with incomplete sets of images, poor image view (i.e., poorly defined or invisible bladder margins), or those excluded by the AI-based tool, were excluded.

All examinations were retrieved from the Picture Archiving and Communication System (PACS). The bladder ultrasound clips were first assessed by an expert sonographer to include only cases that correspond with the inclusion criteria. After obtaining the required data set for the analysis, all pairs (transverse and longitudinal views) were anonymized by the study coordinator and sent to a certified expert ultrasound technician (equivalent to a Registered Diagnostic Medical Sonographer in the United States) for an offline prospective manual measurement of the bladder volumes using digital calipers, which was established as the gold standard. The sonographer was blinded to patient and clinical setting characteristics, as well as the measurements taken by the AI-based tool. The three dimensions of the bladders in the longitudinal and transverse views (i.e., D1, D2, and D3) were acquired. For standardization purposes, the dimensions measured by the expert in the longitudinal view were always marked from top right to bottom left of the image.

The ultrasound images were next automatically assessed by the AI-based module that was applied offline by automated batch processing to all pairs of transverse and longitudinal views and blinded to patient characteristics and the manual tracing results. The calculated volumes by the two methods were then compared.

### 2.4. The Two-Dimensional Ultrasound-Based Bladder Volume Calculation Method

**Figure 1:**
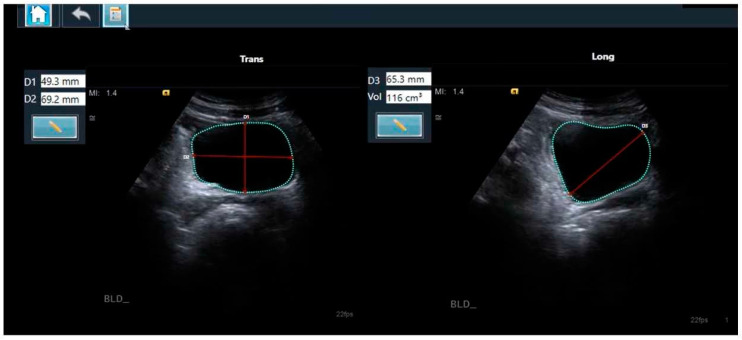
The automated bladder volume and dimension measurements of transverse (**left** panel) and longitudinal (**right** panel) clips, as calculated using the AI-based tool LVivo Bladder, with the prolate ellipsoid method using the three dimensions of the bladder. The red lines represent the individual diameters. The mint-colored shape the automated trace of the outline of the bladder wall.

There are several reported methods for the calculation of bladder volume based on 2D ultrasound images, with the prolate ellipsoid formula most commonly used [26,27]. The prolate ellipsoid method uses the three dimensions of the bladder to calculate the volume based on the mathematical formula V = D1 × D2 × D3 × 0.52, (where V is the volume, D1, D2, and D3 are the respective diameters, and 0.52 is the applied correction factor) (Figure 1) [26].

### 2.5. The AI-Based Module

The AI-based module used was the LVivo Bladder^®^ (DiA Imaging Analysis, Beer Sheva, Israel). This is a commercially available application for fully automated bladder volume measurement, based on 2D ultrasound images. This tool is a decision-support software application that automatically identifies the bladder contours by searching for the dark areas that are regarded as the bladder. The algorithm then determines which area is most likely the bladder, based on edge morphology and heuristics, such as the position, size, and shape of the different areas. The three diameters of the bladder are obtained from the detected contours. The volume is instantly calculated using the prolate ellipsoid method (Figure 1). The manual correction of the bladder border tracings is supported by the application if it is deemed required. In cases of no or minimally recognizable structures that are insufficient for diagnosis, the application will notify that the measurement is not possible.

### 2.6. Sample Size Calculation

Sample size calculations were designed to meet the study’s primary endpoint and were performed using G*Power software (version 3.1.9.4, Heinrich Heine University Düsseldorf, Düsseldorf, Germany). We planned a paired study with a 1:1 ratio comparing the post-void residual bladder volume calculations based on the AI algorithm with the gold standard (an experienced sonographer’s manual measurements). Lacking previous data, we assumed an effect size of 0.3 for the correlation comparing the two measurements. Based on these assumptions, we calculated that the data analyzed from a total of 82 participants (41 pairs of measurements) would suffice to reject the null hypothesis, with a power of 80% at a significance level of 0.05 (two-sided).

### 2.7. The Statistical Analyses

Statistical analysis was performed using the SAS^®^ version 9.3 (SAS Institute, Cary, NC, USA). For the continuous variables, the results were presented as a mean ± SD, and for the categorical variables, the results were presented as percentages. A bladder volume of 200 mL was used as a cutoff, indicating an elevated post-void residual volume (potentially indicating the need for a catheter insertion or more emergently, may raise the suspicion of cauda equina). Continuous values of automatically calculated D1, D2, D3, and the bladder volumes were correlated with the gold standard (an expert sonographer’s manual measurements) by a linear regression analysis using Pearson’s correlation coefficient. The interrater reliability for the diagnosis of an elevated post-void residual volume was evaluated by the kappa coefficient. The strength of agreement by kappa is considered substantial for a kappa-weighted coefficient above 0.61, and almost perfect if it is above 0.81. The agreement between methods was evaluated by a Bland–Altman analysis to calculate the mean difference between the two methods (the bias) and 95% limits of agreement for the mean difference.

The diagnostic value of the LVivo Bladder in identifying patients with and without an elevated post-void residual volume was then tested for its sensitivity, specificity, positive predictive value, and negative predictive value. Reproducibility was determined by calculating both the inter-observer and intra-observer variabilities in a third of the successive examination sets. All tests were two-tailed, and a *p*-value of 5% or less was considered statistically significant.

## 3. Results

A total of 114 complete pairs of transverse and longitudinal images of the bladders of 108 patients were evaluated (108 pre-voiding and 6 post-voiding). Only 3 cases had poor visibility or dropout of the bladder boundaries, and as such, were excluded, leaving a total of 111 pairs of transverse and longitudinal views from 105 patients for inclusion in the analysis. In 39 cases (35.1%), the measured volume was <200 mL.

### 3.1. A Bladder Volume Assessment Comparison: The AI-Based Module vs. the Gold Standard

**Figure 2:**
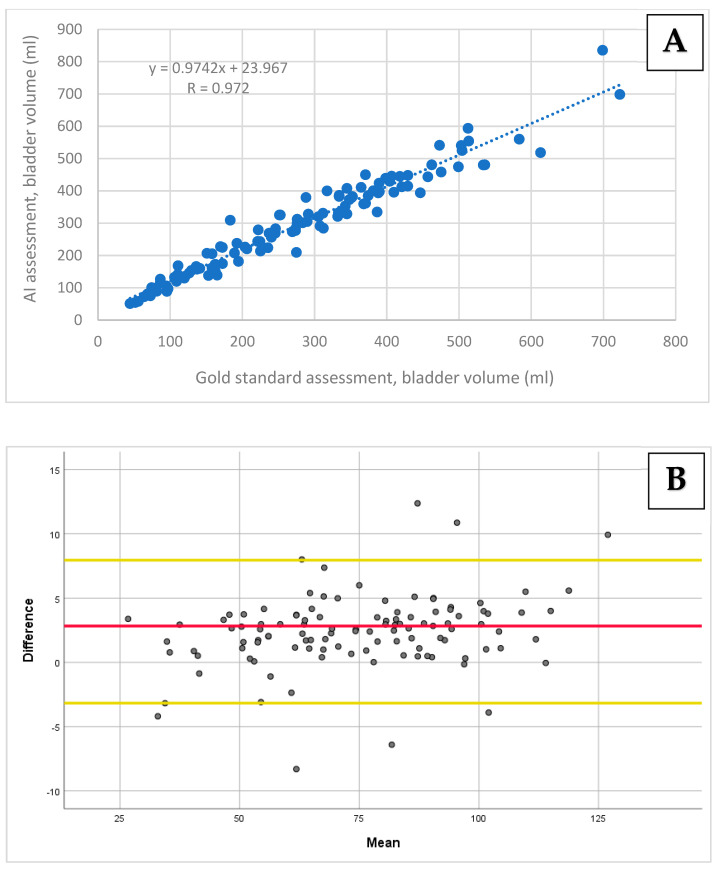
**A** comparison of the bladder volume measurements using the AI-based tool vs. the gold standard: (**A**) The Pearson correlation and (**B**) agreement assessment using the Bland–Altman plot. (**A**) The correlation scatter plot with a regression line and a Pearson correlation coefficient of 0.972 (*p* < 0.001). (**B**) The bladder volume assessment agreement revealed a mean bias of 17.10 (the red line), with the limits of agreement ranging from 86.87 to −52.67 (the yellow lines).

A very strong correlation was obtained between the bladder volume calculated by the AI-based tool and the manual bladder volume measurement, with a Pearson’s correlation coefficient of r = 0.97 (95% CI: 0.96–0.97; *p* < 0.001; Figure 2A). Using the Bland–Altman analysis, the bias and 95% limits of agreement for bladder volume were 17.10 ± 69.77 mL (Figure 2B). An excellent agreement between the manual and automated methods was obtained (kappa = 0.85). The overall agreement was 94%.

### 3.2. A Bladder Diameter (D1, D2, and D3) Measurement Comparison: The AI-Based Module vs. the Gold Standard

**Figure 3:**
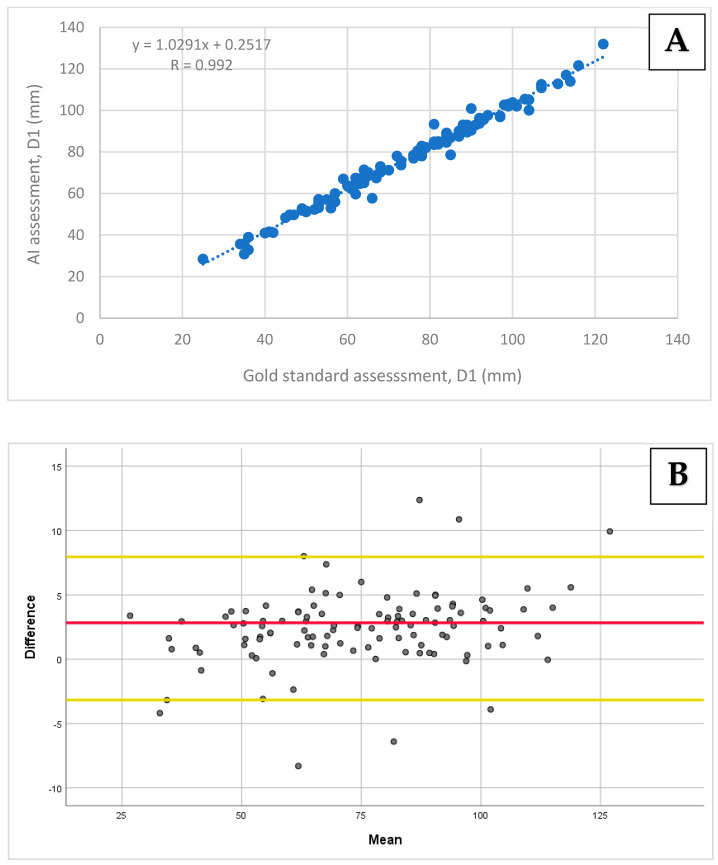
A comparison of the D1 diameter measurements using the AI-based tool vs. the gold standard: (**A**) The Pearson correlation and (**B**) agreement assessment using the Bland–Altman plot. (**A**) A correlation scatter plot with a regression line and a Pearson correlation coefficient of 0.992 (*p* < 0.001). (**B**) The D1 assessment agreement revealed a mean bias of 2.39 (the red line), with the limits of agreement ranging from 7.95 to −3.17 (the yellow lines).

**Figure 4:**
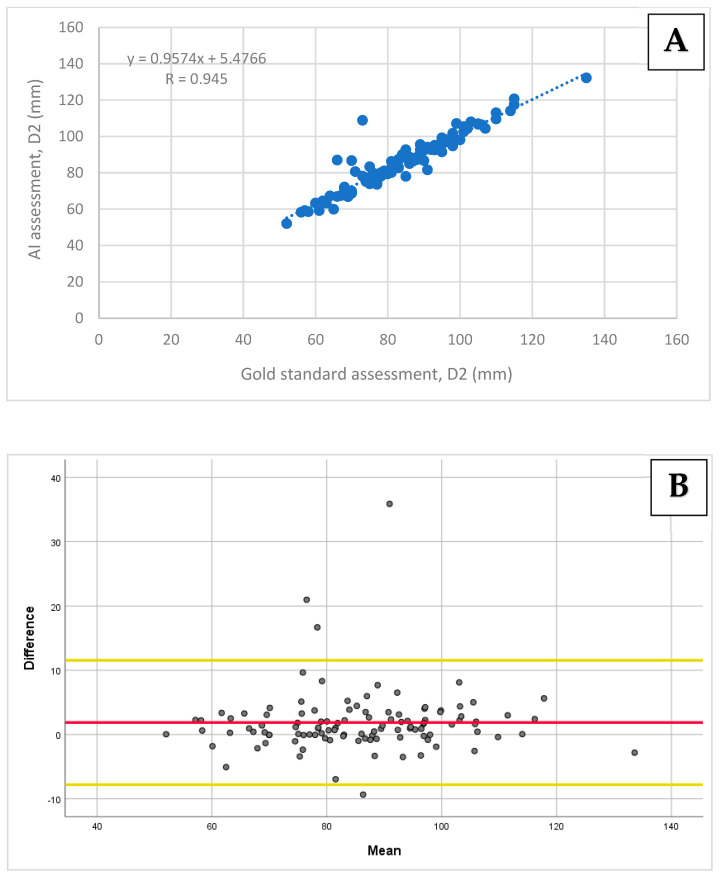
A comparison of the D2 diameter measurements using the AI-based tool vs. the gold standard: (**A**) The Pearson correlation and (**B**) agreement assessment using the Bland–Altman plot. (**A**) A correlation scatter plot with a regression line and a Pearson correlation coefficient of 0.945 (*p* < 0.001). (**B**) The D2 assessment agreement revealed a mean bias of 1.87 (the red line), with the limits of agreement ranging from 11.55 to −7.81 (the yellow lines).

**Figure 5:**
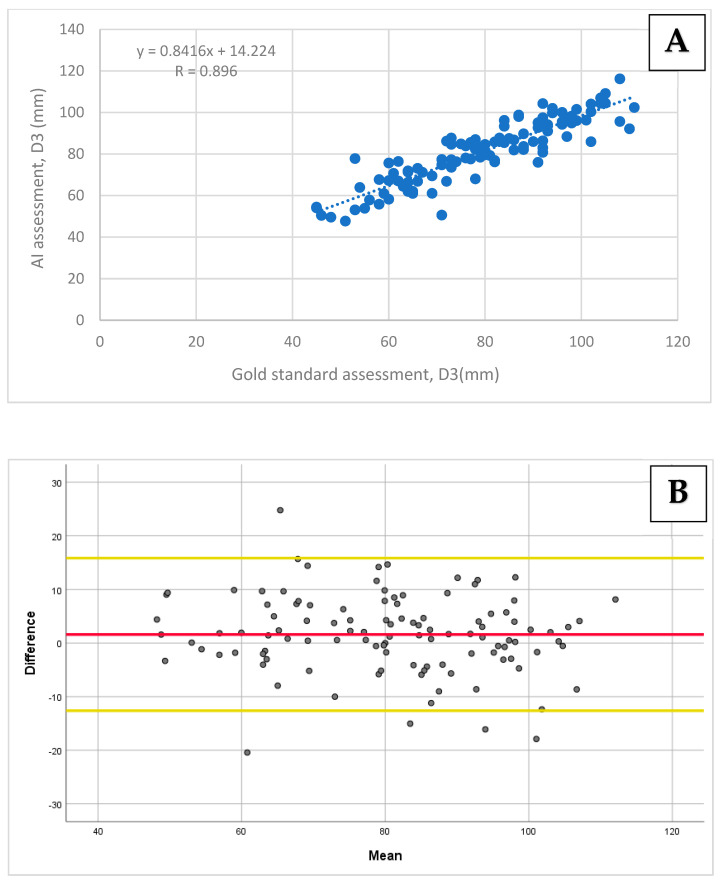
**A** comparison of the D3 diameter measurements using the AI-based tool vs. the gold standard: (**A**) The Pearson correlation and (**B**) agreement assessment using the Bland–Altman plot. (**A**) A correlation scatter plot with a regression line and a Pearson correlation coefficient of 0.896 (*p* < 0.001). (**B**) The D3 assessment agreement revealed a mean bias of 1.61 (the red line), with the limits of agreement ranging from 15.83 to −12.62 (the yellow lines).

A very strong correlation was found between the AI-based assessment of D1 and D2 compared to the gold standard, with Pearson’s correlation coefficients of r = 0.99 (95% CI: 0.98–0.99; *p* < 0.001) and r = 0.95 (95% CI: 0.93–0.97; *p* < 0.001), respectively (Figure 3A and Figure 4A). A strong correlation was obtained for D3 when comparing the two methods, with a Pearson’s correlation coefficient of r = 0.90 (95% CI: 0.84–0.92; *p* < 0.001; Figure 5A).

The biases and limits of agreements were calculated for all diameters using the Bland–Altman analysis. For D1 and D2, the biases and 95% limits of agreement were 2.39 ± 5.56 mm and 1.87 ± 9.68 mm, respectively (Figure 3B and Figure 4B). For D3, the bias and 95% limits of agreement were 1.61 ± 14.22 mm (Figure 5B).

### 3.3. The Sensitivity and Specificity Analyses

The sensitivity and specificity for the diagnosis of an elevated post-void residual volume (≥200 mL) using the AI-based tool were 0.82 and 1.0, respectively. The NPV and PPV were 0.911 and 1.0, respectively.

### 3.4. The Inter- and Intra-Observer Variabilities

The intra-observer agreement was tested on 38 cases. For the manual measurements of the total bladder volume, the intra-observer correlation was r = 0.98. An excellent correlation of r = 0.98, r = 0.98, and r = 0.93 was obtained for the manual measurement of D1, D2, and D3, respectively. For the AI-based module, the measurement values remained the same, with a correlation of r = 1 obtained for all measurements.

The inter-observer biases and 95% limits of agreement were obtained for the manual measurements of the total volume, revealing the bias and limits of agreement of 3.2 ± 54.4 mL. For D1 and D2, the bias and 95% limits of agreement were −1.2 ± 5.6 mm and −0.15 ± 3.2 mm, respectively. For D3, the bias was small, with wider limits of agreement of 1.4 ± 12.2 mm.

## 4. Discussion

The incorporation of POCUS in various settings, including among different medical and nursing personnel, is now ubiquitous, with a myriad of diagnostic and procedural applications [28,29,30,31]. Bladder volume measurement is merely a single application that is being incorporated daily in these settings [32]. The present study showed that there was an excellent correlation between the bladder volumes calculated by a fully automated algorithm and the manual measurements taken by experienced sonographers. In five cases, the absolute difference between the volumes was >80 mL. In all of these cases, the manually estimated volumes were above 280 mL. A small difference in the measurements of diameters may result in large differences in volume (since the volume is calculated as a multiplication of three diameters). However, these differences did not have an impact on the overall categorical agreement between the methods. Although the validation was conducted by comparing manual measurements with the AI-based fully automated measurements, manual correction is supported by the LVivo Bladder application, and if required, can be easily applied. Also, the present study focused mostly on the pre-voiding bladder assessment, given the clinical relevance and interest in assessing high bladder volumes and providing proper validation of a wide volume range.

There was also an excellent intra-observer agreement. The results found very high correlations for all three diameters, as well as for the volume. Small biases and limits of agreement were obtained for D1 and D2. For D3, the bias was small, with wider limits of agreement, indicating a greater variability in the measurement of this diameter. Similar results were obtained for D3 when comparing the automated and manual measurements, which could be explained by the variability in repeated measurements of D3. For the volume, the intra-observer bias was small, but there were differences between the repeated measurements, similar to the differences obtained when comparing the AI-based volumes to the manual assessments, reflecting the “natural” variability in the manual measurements.

Similar to this application, AI is increasingly being combined with POCUS. Many of these applications exist in the field of cardiac POCUS. It was shown that medical students who underwent a six-hour course that focused on image acquisition could accurately assess left ventricular ejection fractions using an AI-based tool, and that this assessment independently predicted a 1-year mortality and cardiovascular-related readmission, and was associated with unfavorable in-hospital outcomes [33,34]. A Japanese study from two hospitals also validated an AI program that assessed LVEF using a handheld ultrasound device [35]. AI is also being incorporated into the evaluation of cardiac output, which is critical for a sick patient in the emergency department or intensive care unit [36]. Nurses have also been shown to be able to identify patients with heart failure using an AI-based device. Also, in the field of cardiology, AI algorithms are being used to detect cardiomyopathies [37]. AI is also being incorporated in lung applications. This has even been extended to the patients themselves. In a recent study, dialysis patients performing a self-ultrasound with an AI application that counts B-lines were able to monitor for pulmonary congestion [38].

Bladder ultrasound is an area where AI can significantly enhance the accurate assessment of urinary volumetry. Bladder scanners are dedicated single-purpose ultrasound devices, often operated by less-experienced medical and nursing personnel, that use a three-dimensional volumetric probe to measure bladder volume. They provide quick automatic measurements of the bladder volume and are easy to operate [39]. Despite their wide uses and advantages, bladder scanners have limitations. They are expensive tools, mostly utilized for a single clinical purpose, and usually provide a bladder volume estimation without a direct visual of the bladder (especially with early bladder scan models), thus failing to accurately distinguish between the bladder and different structures or collections, including pelvic cysts, ascites, and hemorrhages [40,41]. Bladder scanners are also less reliable in pregnant patients, children, and neonates, patients requiring peritoneal dialysis, with morbid obesity, and after bladder augmentation surgeries [23,42,43,44]. Bowel loops filled with fluids can also be mistaken for bladder content and may present false measurements [45].

It was shown that, if used correctly, traditional 2D ultrasound imaging provides more accurate results than bladder scanners [46]. Bladder volume evaluation by 2D ultrasound devices requires the operator to scan the patient from two views and manually mark the three diameters of the bladder on the screen. This is a time-consuming and subjective procedure that requires experience to be performed correctly [26]. Because there are no clear guidelines on how to manually measure these three dimensions (especially the diameter in the longitudinal view) when using a 2D ultrasound device in a POCUS environment, the bladder volume is often roughly evaluated by a visual estimate of the scanned images based on the operator’s previous experience. While bladder scanners are delegated to a single task, there is a growing use of 2D ultrasound devices, portable and handheld, in POCUS settings to visualize and evaluate various systems across multiple medical fields, including the cardiovascular, respiratory, and abdominal, and to perform various guided procedures [47,48]. These devices are multipurpose and their use in the POCUS environment is expanding rapidly, especially following the recent COVID-19 pandemic. Given their advantages, including being wireless, internally battery-operated, having a touch screen, can be assigned to specific departments, and are free from unnecessary elements that make them difficult to clean, they may, thus, dramatically reduce the risk of cross-infection transmission [49]. Nonetheless, handheld ultrasound device utilization can involve multiple challenges, including having a small screen size, a limited battery time, suboptimal imaging quality, limited advanced measurements, and the possibility of reporting ambiguous findings requiring confirmation by a high-end device [50].

An algorithm that enables an automated bladder volume calculation is a great advantage in the novice user’s POCUS environment, since the same ultrasound device that is already used in the department could be used, for example, by the physician’s assistant in the emergency department or nurse in triage. In addition, it could be of use in handheld scanners that are gaining in popularity [51,52,53]. The ability to incorporate a user-friendly automated algorithm for accurate bladder volume measurement with a simple handheld device may allow nurses in triage or less-experienced medical personnel to rapidly diagnose a clinically significant post-void residual volume. Additionally, an AI-based module can serve as a validation tool for the confirmation of quantitative bladder volume assessments conducted by novice users.

While POCUS for the bladder are most often used to measure bladder volume in patients presenting with urinary retention and suspected prostatic hypertrophy, a more emergent application is to rapidly identify the patients presenting clinical symptoms suggestive of cauda equina syndrome. This is a rare, time-critical diagnosis in the emergency department, with a poor prognosis if missed, or if operative repair is delayed [54]. This is especially important because cauda equina syndrome may present a wide range of signs and symptoms that are not necessarily specific [55]. It is challenging to diagnose cauda equina syndrome, as no single clinical finding or combination of findings is sensitive or specific enough to predict its presence [56]. Bladder dysfunction in the form of urinary retention or overflow incontinence is a late presentation of cauda equina syndrome and may be the only presenting sign of this pathology [57]. Assessing bladder volume by ultrasound to detect post-void residual volume in patients with suspected cauda equina syndrome may lead to a rapid diagnosis and treatment.

We have shown that only 3 cases (2.6%) out of a total of 114 included in our study had poor imaging quality precluding analysis by the AI-based tool when acquired by an experienced sonographer. Similar rates of low-quality imaging were presented in a recent prospective study assessing the acquisition accuracy among five medical students trained in different POCUS applications, including bladder volumetry. The study had demonstrated that 51/53 bladder volume scans acquired from pediatric patients were graded as acceptable (96.2%; 95% CI 87.3–99.0%) using the American College of Emergency Physician’s Emergency Ultrasound Standard Reporting Guidelines’ 5-point quality assurance (QA) scale [58].

### Limitations

This study was conducted on ultrasound video clips of urinary bladders with their prospective offline measurements. Thus, ‘hard’ clinical outcomes, such as the need for an indwelling urinary catheter insertion, the rate of acute kidney injury, or urinary tract infection, were not reported or evaluated. Also, this study was restricted to a single site and may be subjected to the relevant confounders that may limit the generalizability of the findings. Furthermore, the imaging ultrasound clips used in the assessment of the AI-based tool were acquired by experienced sonographers, and as such, may not reflect the same accuracy when acquired by novice physicians or nurses with a possible suboptimal image quality or bladder foreshortening. Also, the ultrasound devices that were used for the imaging acquisition were relatively more expensive and had a higher resolution and image quality than the average handheld ultrasound device, and as such, the results of the present study may not apply to all POCUS settings. However, as mentioned above, bladder ultrasound is fairly simple, and when tested with medical students and novice users, the acquisition rate of bladder images of an acceptable quality was similar to those reported in the present study. The next research step will further assess the impact of this AI-based application by medical and nursing clinicians’ decision making in the context of a real-life clinical workflow. Of note, we did not validate the technician’s measurements used as the gold standard, as it would involve routine diameter measurements that were proven to be accurate and were validated for their reproducibility, comparisons between different operators, as well as indwelling urinary catheter insertions [23].

## 5. Conclusions

This novel AI-based method demonstrates an excellent correlation with manual bladder volume measurements by experienced sonographers, and its ease of use can be used as a decision-support tool in the POCUS environment by novice medical personnel. It can be easily installed on many of the multiuse wheel-based or handheld ultrasound devices that are now ubiquitous in various settings, including the emergency department. It can also be easily applied by novice users for both pre- and post-void volume assessments. This may aid in the rapid diagnosis of urinary outflow obstruction and in the time-critical diagnosis of cauda equina syndrome.

As POCUS become increasingly integrated with medical routines and clinical practices across various settings, there should be a diminishing reliance on urinary catheterization solely for the purpose of assessing bladder volume. Medical and nursing leaders may consider generalizing the study results in their future incorporation of AI-based tools for the augmentation of ultrasound assessment of bladder volume in various clinical settings and healthcare systems for the broader patient population, including in the emergency department, the medical ward, and in the clinic. This tool can be used especially in programs that utilize POCUS for rapid clinical assessment as a decision-support tool before urinary catheter insertion. While not studied in the current research, future studies may contemplate employing this automated tool as a secondary opinion, particularly among novice users and nurses tasked with assessing bladder volume for educational purposes.

## Data Availability

The data presented in this study are available upon request from the corresponding authors.
